# Inferior Vena Cava Thrombosis and Pulmonary Embolism in a Patient With Behcet Disease: A Rare Presentation

**DOI:** 10.7759/cureus.44184

**Published:** 2023-08-27

**Authors:** Muhammad Hamza Mushtaq, Osama Ali Khan, Hasnat Khan, Muhammad Tabish Ikram, Muhammad Danyal Tariq

**Affiliations:** 1 Internal Medicine, Lady Reading Hospital, Peshawar, PAK; 2 Internal Medicine, Khyber Teaching Hospital, Peshawar, PAK; 3 Medicine, Hayatabad Medical Complex Peshawar, Peshawar, PAK; 4 Internal Medicine, Khyber Medical College, Peshawar, PAK

**Keywords:** behcet syndrome, venous sinus thrombosis, persistent oral ulcers, genital ulcers, uveitis, pulmonary embolism, right atrial thrombus, inferior vena cava thrombosis, vasculitis

## Abstract

Behcet syndrome is a systemic vasculitic syndrome. Vascular involvement in Behcet syndrome affects both arterial and venous vascular systems, contributing to significant morbidity and mortality. However, diagnosing vascular lesions can be challenging due to their resemblance to common vascular diseases, leading to potential misdiagnoses. This case report emphasizes the importance of recognizing atypical manifestations of this disease to ensure a prompt and accurate diagnosis. This case report describes a unique presentation of Behcet syndrome in a 23-year-old male patient who presented with per rectal bleeding, abdominal distension, right quadrant abdominal pain, pleuritic chest pain, and fever. The patient also reported a history of recurrent oral and genital ulcers, skin lesions, and a previous episode of dural venous sinus thrombosis. Extensive investigations revealed the involvement of the inferior vena cava and right hepatic vein, representing an atypical vascular manifestation of Behcet syndrome. Prompt diagnosis by a multidisciplinary team led to appropriate treatment with cyclophosphamide and steroids, resulting in the resolution of vascular thrombosis. In this particular case, the patient presented with involvement of the inferior vena cava and right hepatic vein, a rare and unusual manifestation of the disease. This case highlights the diverse nature of vascular complications in Behcet syndrome and underscores the importance of considering this diagnosis in patients with unexplained vascular abnormalities. Overall, this case report highlights the importance of considering Behcet syndrome in the differential diagnosis of patients with unexplained vascular manifestations. It also emphasizes the need for a comprehensive clinical evaluation and collaborative approach to ensure timely and effective management.

## Introduction

Behcet syndrome, or Behcet disease, is an uncommon multi-system disorder with a relapsing and remitting nature. It can manifest with skin, urogenital, gastrointestinal, neurological, and vascular system involvement [1]. Behcet syndrome predominantly affects male individuals more than females. A more severe disease course is commonly associated with the male gender and an earlier onset of diagnosis, particularly in younger individuals [2]. Behcet syndrome occurs worldwide but exhibits a higher prevalence in regions along the ancient Silk Road, including the Middle East, Mediterranean, and Far East. With this geographical distribution, the condition has earned the name "the Silk Road disease" [3]. The worldwide prevalence is estimated to be approximately 10.3 cases per 100,000 individuals. Notably, Turkey has the highest reported prevalence, reaching up to 420 cases per 100,000 population [4-6]. Vascular involvement in Behcet syndrome is distinctive as it affects both the arterial and venous vascular systems of various calibers, making it a significant contributor to morbidity and mortality in affected individuals [7]. The clinical presentation of vascular lesions in Behcet syndrome often poses a diagnostic challenge, as they closely resemble manifestations of common vascular diseases. This can contribute to missed or misdiagnosis, potentially leading to serious consequences. Venous disease in Behcet syndrome often presents as lower-extremity venous thrombosis [8]. However, our reported case is unique as it presents with the involvement of the inferior vena cava, highlighting an atypical manifestation of the disease.

## Case presentation

**Figure 1:**
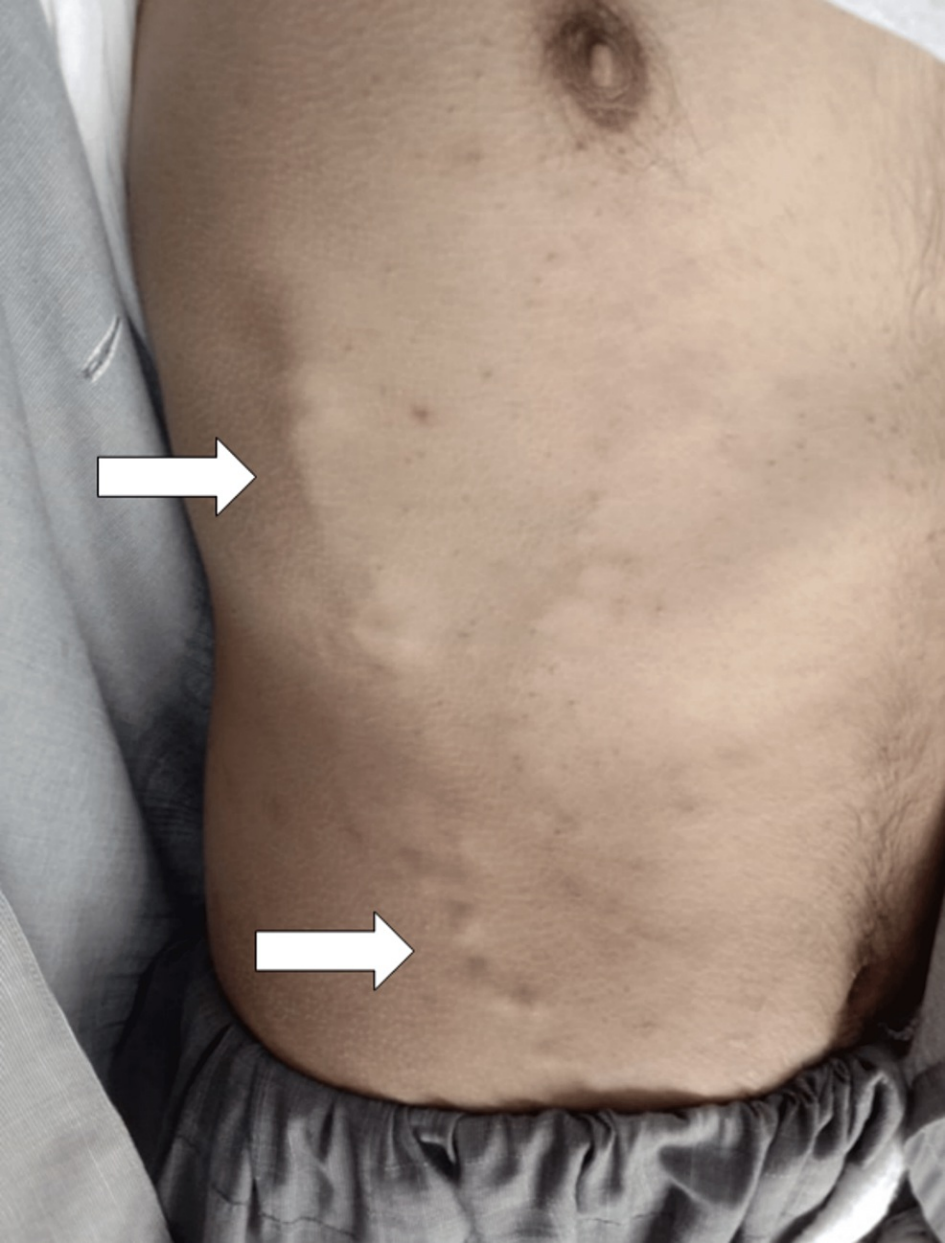
Superficial collateral veins over the right chest wall and right flank (white arrows)

A 23-year-old male patient presented to the outpatient department of Khyber Teaching Hospital, Peshawar, Pakistan, with the chief complaints of per rectal bleeding, progressive abdominal distension, right quadrant abdominal pain, pleuritic chest pain, and fever for the past 10 days. The patient also complained of recurrent oral and genital ulcers and occasional vision disturbances for 10 years. He also gave a history of multiple papular skin lesions on the forearms and shins, especially after drawing blood from the forearm veins. On inquiring about his medical history, we learned that he was admitted in 2016 for severe headaches and vomiting. On his workup, MR venography revealed dural venous sinus thrombosis. Subsequently, his thrombophilia screen (protein C, protein S, antithrombin III, and factor V Leiden) was sent, which came back negative, and he was discharged on warfarin. As the patient had now developed per rectal bleeding, warfarin was appropriately stopped. On general physical examination, the patient was clinically anemic, had aphthous ulcers in the oral cavity, and had multiple erythematous lesions on the forearms and shins. Abdominal examination showed distension and superficial collateral veins on the right chest wall and right flank (Figure 1). On palpation, the abdomen was tender in the right quadrant, and ascites with a fluid thrill could be appreciated. Chest auscultation revealed decreased breath sounds bilaterally. The remaining examination was inconclusive. Baseline investigations, viral profile, blood cultures, serum albumin, antinuclear antibody (ANA), erythrocyte sedimentation rate (ESR), chest X-ray, ascitic tap for fluid r/e, Doppler ultrasound of the abdomen with pelvic ultrasound, and echocardiogram were ordered. In addition, ophthalmologic consultation was sought for fundoscopy.

His complete blood counts showed hemoglobin levels of 8.2 g/dL, a total leukocyte count of 17,760 per μL with neutrophilic predominance, and platelets of 266,000/μL with a hematocrit of 26.8%. His liver function tests revealed elevated alanine transaminase (ALT) and alkaline phosphatase (ALP) levels of 154 U/l and 344 U/l, respectively. Furthermore, the patient had normal renal function tests and a lipid profile. The patient also had a raised reticulocyte count of 3%. He had an international normalised ratio (INR) of 3.42 as he was on warfarin. Urinalysis showed 1+ proteins but no pus cells or red blood cells (RBCs). His ESR was 108 mm/first hour, and ANA came back negative. Blood cultures did not show the growth of any microorganism. The viral profile was negative for hepatitis B surface antigen (HBsAg), anti-hepatitis C virus (HCV), and anti-human immunodeficiency virus (HIV) antibodies. Serum albumin was 2.7 g/dl. His D-dimer levels were also raised to 2462 ng/ml (Table 1).

**Table 1:** Laboratory results of the patient with reference ranges. ALT, alanine transaminase; ALP, alkaline phosphatase; INR, international normalized ratio; ESR, erythrocyte sedimentation rate; ANA, antinuclear antibodies; HBsAg, hepatitis B surface antigen; HCV, hepatitis C virus; HIV, human immunodeficiency virus; ELISA, enzyme-linked immunosorbent assay; CRP, C-reactive protein

Parameter	Value	Reference range
Total leukocyte count	17,760/μL	4,000-10,000/μL
Hemoglobin	8.2 g/dL	13.0-17 g/dl
Platelet count	266,000/μL	150,000-300,000/μL
Hematocrit	26.8%	40-54%
ALT	154 U/L	7-55 U/L
ALP	344 U/L	44-147 U/L
Reticulocyte count	3%	0.5-2.5%
INR	3.42	1
ESR	108mm/1st hour	0-15mm/1st hour
ANA	Negative	
HBsAg (ELISA)	Negative	
Anti-HCV (ELISA)	Negative	
Anti-HIV (ELISA)	Negative	
Serum albumin	2.7 g/dl	3.4-5.4 g/dl
D-dimer	2462 ng/ml	<500 ng/ml
CRP	153 mg/L	<5.0 mg/L

**Figure 2:**
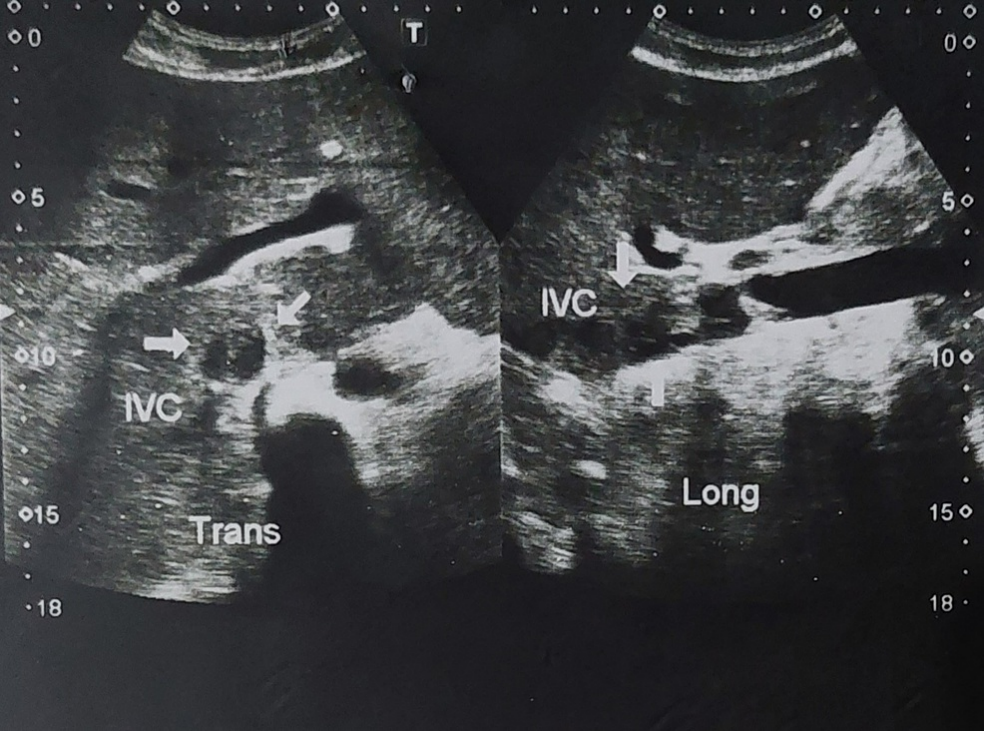
Ultrasound abdomen and pelvis showing inferior vena cava thrombus

The patient’s ascitic fluid analysis revealed a leukocyte count of 514/cmm, of which 70% were lymphocytes and 30% were neutrophils. The serum-ascites albumin gradient (SAAG) level was 1.23, which pointed toward portal hypertension as it was greater than 1.1 g/dL. There were no atypical cells in the ascitic fluid (Table 2). Ultrasound abdomen and pelvis revealed hepatomegaly with moderate ascites, inferior vena cava (IVC), and right hepatic vein thrombosis (Figure 2). The echocardiography report revealed an echogenic mobile mass, soft in appearance and irregular in shape as seen in the right atrium protruding into the right ventricle with its attachment near IVC. IVC was non-collapsing and had a thick mass. These findings were suggestive of an IVC thrombus extending into the right ventricle through the right atrium. Fundoscopy of the patient showed bilateral vitreous cells suggestive of uveitis. A CT pulmonary angiogram was ordered as the patient had a right atrium thrombus protruding into the right ventricle and was complaining of pleuritic chest pain with hemoptysis to rule out pulmonary embolism, which subsequently revealed filling defects in the segmental branches of pulmonary arteries bilaterally in the lower lobes with basal consolidation, suggestive of pulmonary thromboembolism.

**Table 2:** Ascitic fluid analysis with the SAAG ratio TLC, total leukocyte count; RBCs, red blood cells; LDH, lactate dehydrogenase SAAG, serum-ascites albumin gradient

Parameter	Value	Reference range
Amount	1.5 ml	
Color	Yellow	Straw colored
Appearance	Slightly turbid	Clear
Coagulum	Present	Absent
TLC	226/cmm	100/cmm
RBCs	+	None seen
Polymorph	60%	2%
Lymphocytes	40%	98%
Protein	3.59 g/dl	4.1%
Glucose	106 mg/dl	103 mg/dl
LDH	140 U/L	
Albumin	1.47 g/dl	
Serum albumin	2.7 g/dl	
SAAG	1.23 d/dl	

As the patient’s thrombophilia screen was previously negative, he was suspected of having underlying vasculitis as a cause of recurrent thrombosis, and the rheumatology team was subsequently involved. The rheumatology team, after taking a detailed history and doing the relevant physical examination, diagnosed the patient with Behcet syndrome in light of the recurrent oral and genital ulcerations, vision abnormalities, recurrent thrombosis, and positive pathergy test as the diagnosis of Behcet disease is clinical.

The patient was subsequently treated by the rheumatology team with cyclophosphamide 1 gm IV in the hospital as he had developed thrombotic complications of Behcet disease. He was discharged on tab colchicine 0.5 mg three times daily for the prevention of oral and genital ulcers. In addition, the patient was started on tab prednisone 5 mg, four tablets twice daily for the first two weeks (40 mg daily), followed by three tablets twice daily (30 mg daily) for the next two weeks. The dose was to be tapered off over the next four weeks. On the first one-month follow-up visit, tab colchicine was stopped as there were no new oral or genital ulcers. 

Repeat echocardiography and Doppler ultrasound of the abdomen were performed one month later, which showed resolution of the IVC and right atrial thrombus.

## Discussion

As per the International Study Group (ISG) criteria, Bechet disease is defined as recurrent oral ulcers with two of the following: recurrent genital ulcers, eye lesions, skin lesions, or a positive pathergy test [9]. Our patients had oral ulcers with eye and skin lesions and positive pathergy tests. Behcet disease is a systemic vasculitis, and the pathogenesis involves relapsing-remitting episodes of acute inflammation of vessels [10].

Although the exact cause is unknown and is thought to be multifactorial, there is a strong link in patients with HLA-B*51 of the major histocompatibility complex (MHC) class 1 region, with almost 70-fold more chance of getting the disease in patients with HLA-B51 allele than those who do not [11]. Vessel wall inflammation and subsequent thrombus formation seem to play a role in the underlying pathology, and T1 helper cell response is predominant in this inflammation [12].

The common clinical feature of patients with Bechet disease is mucocutaneous ulcers, but it can also involve other systems, such as gastrointestinal, neurological, and vascular [13]. The oral and ocular symptoms of the disease are the most common manifestation, with a frequency of more than 80% [14].

Vascular involvement of the disease also occurs and is a major cause of mortality and morbidity, but it is not as common as cutaneous symptoms. In one study of the Turkish population with Bechet disease, the prevalence of vascular disease was 14.3% [15]. Both venous and arterial vessel involvements can occur, with venous more common than arterial. The entire venous system, from superficial to deep veins, is at risk of developing thrombosis. Arterial vessel inflammation can present either as hemorrhage, stenosis, aneurysm formation, or thrombus. IVC occlusion (15% of patients), hepatic vein thrombosis (occur commonly with vena cava thrombus), dural sinus venous thrombosis (very rare, only 2.5% incidence), and other venous obstructions, in addition to the more common superficial and deep vein thrombosis, are sometimes the early signs of Bechet disease. Involvement of pulmonary and hepatic veins is associated with high morbidity and mortality [16].

Bechet disease usually has a delay in diagnosis of several years, as in the case of our patients, in which venous thrombosis separated in time and space, misleading to a diagnosis of thrombophilia disorders and administration of anticoagulation. A thorough medical history and clinical exam led to the diagnosis, and a multidisciplinary team approach led to a prompt therapeutic intervention. In Bechet disease, conditions associated with higher mortality, such as pulmonary embolism and hepatic vein thrombus, require an aggressive medical approach with drugs, including cyclophosphamide and steroids [17].

## Conclusions

This unique case of Behcet syndrome highlights the complexity and diagnostic challenges of this rare multi-system disorder. The atypical vascular involvement underscores the need for heightened awareness among healthcare professionals to recognize and accurately diagnose such manifestations. A collaborative approach and thorough evaluation are crucial to ensuring timely and effective management. Continued research and knowledge-sharing will further enhance our understanding and treatment of this enigmatic condition.
